# Remote‐Controlled Release of Nitric Oxide by Plasmonic Waveguide Coated With Metal–Organic Frameworks

**DOI:** 10.1002/smll.74836

**Published:** 2026-07-31

**Authors:** Tomoko Inose, Javier Lopez‐Cabrelles, Javier Troyano, Shun Tokuda, Elí Sánchez‐González, Nobuaki Takahashi, Yasuo Mori, Kenji Hirai, Hiroshi Uji‐i, Shuhei Furukawa

**Affiliations:** ^1^ Institute For Integrated Cell‐Material Sciences (WPI‐iCeMS) Kyoto University Sakyo‐ku Kyoto Japan; ^2^ The HAKUBI Center for Advanced Research Kyoto University Sakyo‐ku Kyoto Japan; ^3^ JST‐PRESTO Saitama Japan; ^4^ Department of Synthetic Chemistry and Biological Chemistry Graduate School of Engineering Kyoto University Nishikyo‐ku Kyoto Japan; ^5^ Research Institute For Electronic Science (RIES) Hokkaido University Sapporo Japan; ^6^ KU Leuven Department Chemie Heverlee Belgium

**Keywords:** metal–organic frameworks, nanowire, nitric oxide, plasmonic waveguide

## Abstract

Spatiotemporal control of photochemical reactions in living cells is a powerful approach for the precise manipulation of cellular functions and the interrogation of biological systems. However, conventional methods requiring direct light exposure often induce cellular damage due to the generation of reactive oxygen species and/or photothermal effects. Here, we present a novel intracellular delivery method using the plasmonic waveguiding effect, which enables the release of bioactive molecules within a single live cell without subjecting the target cells to direct light exposure. This is achieved by remotely inducing photo‐cleavage reactions via the propagation of surface plasmon polaritons (SPPs), which are generated by light irradiation at a spatially separated coupling point and guided to the target site. We demonstrate the efficacy of this platform by delivering nitric oxide (NO) gas into an individual single live cell, confirming its biological relevance by monitoring transient changes in intracellular calcium concentration. This novel platform provides a precise, time‐controlled means of intracellular molecular delivery, offering a new frontier for minimizing invasive single‐cell surgery and signaling studies.

## Graphical abstract

Remote‐controlled intracellular delivery is achieved using a plasmonic waveguide coated with a photoresponsive NO‐releasing MOF. Surface plasmon polariton propagation remotely induces photocleavage and localized nitric oxide release in a target single live cell without direct light exposure, while biological relevance is demonstrated through transient intracellular calcium responses.

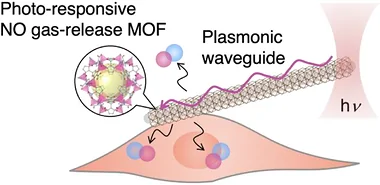

**Abbreviations**
2‐nIm: 2‐nitroimidazole
AC: alternating current
AgNWs: Ag nanowires
AuAgNWs: gold‐etched silver nanowires
DAF‐FM: diaminofluorescein‐FM
EDS: energy‐dispersive X‐ray spectroscopy
GRR: galvanic replacement reaction
HASMC: human aortic smooth muscle cell
MOF: metal‐organic frameworks
NIR: near‐infrared
NO: nitric oxide
NOF‐1: NO‐release metal‐organic framework
PMMA: poly(methacrylic acid)
PVP: polyvinylpyrrolidone
ROS: reactive oxygen species
SEM: scanning electron microscopy
SPPs: surface plasmon polaritons
TRPC5: transient receptor potential channel 5.
XRD: X‐ray diffraction

## 1 Introduction

Optical technologies serve as fundamental tools for imaging cellular events and controlling biological functions [1, 2, 3, 4, 5, 6, 7]. Their high degree of spatiotemporal controllability enables the detailed observation and manipulation of living systems at specific subcellular locations and precise time points. These advantages are further amplified when optical technologies are integrated with photo‐responsive molecular tools, which allow precise intervention in living systems through locally induced photochemical reactions. For example, photocleavable molecular scaffolds have been successfully employed to activate intracellular prodrugs and enzymes [8, 9, 10, 11, 12, 13]. Similarly, molecular switches based on photoisomerization can modulate ligand binding or ion channel gating, while photoinduced redox transfer can alter catalytic states [14, 15, 16]. These photo‐responsive molecules are also useful for intracellular molecular delivery by uncaging cargo or transiently opening carrier structures [17, 18, 19, 20, 21]. Consequently, the application of diverse photo‐responsive molecules to induce well‐defined photochemical reactions has become an essential optical strategy for interrogating live‐cell functions.

**FIGURE 1:**
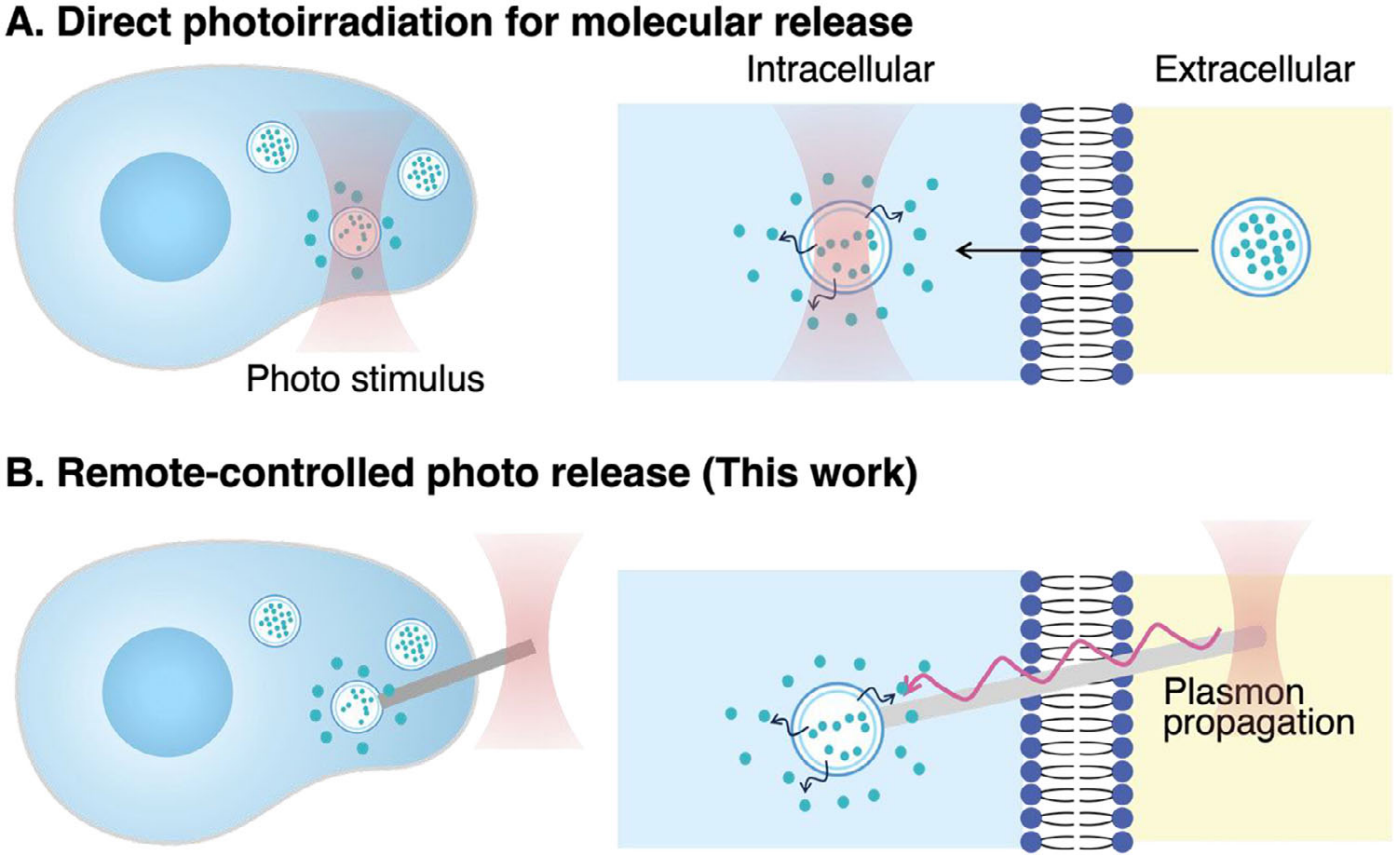
Release of molecules inside a cell. (A) Illustration of the direct photoirradiation of a target cell. (B) Remote‐controlled local release of molecules by plasmonic waveguide.

Despite these advancements, most existing systems still rely on direct photo‐irradiation to initiate photochemical reactions (Figure 1A). Such illumination could cause significant cytotoxicity in live cells, particularly under prolonged or intense exposure. This process often induces the generation of reactive oxygen species (ROS) from introduced photo‐reactive molecules and/or endogenous photo‐responsive biomolecules, leading to oxidative stress, membrane disruption, and eventual cell death [22, 23]. Furthermore, photothermal effects induced by high‐intensity light irradiation could cause localized heating, potentially damaging cellular structures or perturbing biological functions [24]. These unintended side effects remain an important consideration when applying photoresponsive systems to minimally invasive and spatially precise cellular manipulation.

To address these challenges, we present a novel plasmonic nanowire‐based single live‐cell endoscopy approach [25, 26, 27, 28, 29]. This method remotely induces photochemical reactions and controls molecular release within a single live cell without directly exposing the target cell to an external light stimulus (Figure 1B). Plasmonic nanowires exhibit a unique waveguiding effect: surface plasmon polaritons (SPPs), generated via photoexcitation, propagate along the nanowire over micrometer distances [30, 31]. This property allows the spatial decoupling of the focused intense far‐field excitation light from the localized signal‐detection point or reaction site. In our previous studies, we demonstrated that this plasmonic waveguiding effect can be used for remote excitation in Raman and fluorescence spectroscopy [25, 31, 32]. In addition, we developed a method to precisely insert a silver nanowire with a diameter of approximately 120 nm and a length of several tens of micrometers into a specific site of a single live cell with minimal physical disruption, thereby applying this remote excitation strategy to the cellular environment [25].

In this study, we have successfully extended this platform to induce remote control of photo‐cleavage reactions at the single‐cell level. As a proof‐of‐concept, the nanowire is functionalized with photo‐reactive metal–organic frameworks (MOFs) designed to release nitric oxide (NO) upon photo‐stimulation. We demonstrate the controlled intercellular release of NO and validate its biological relevance by observing a transient increase in the intracellular calcium ion, mediated by NO‐sensitive plasma membrane channel proteins. This novel platform overcomes the limitations of conventional photo‐stimulation by eliminating direct irradiation, thereby mitigating phototoxicity and enabling highly localized, remote‐controlled intracellular delivery.

## 2 Results and Discussion

### 2.1 Design, Synthesis, and Characterization of MOF‐Coated Nanowires

**FIGURE 2:**
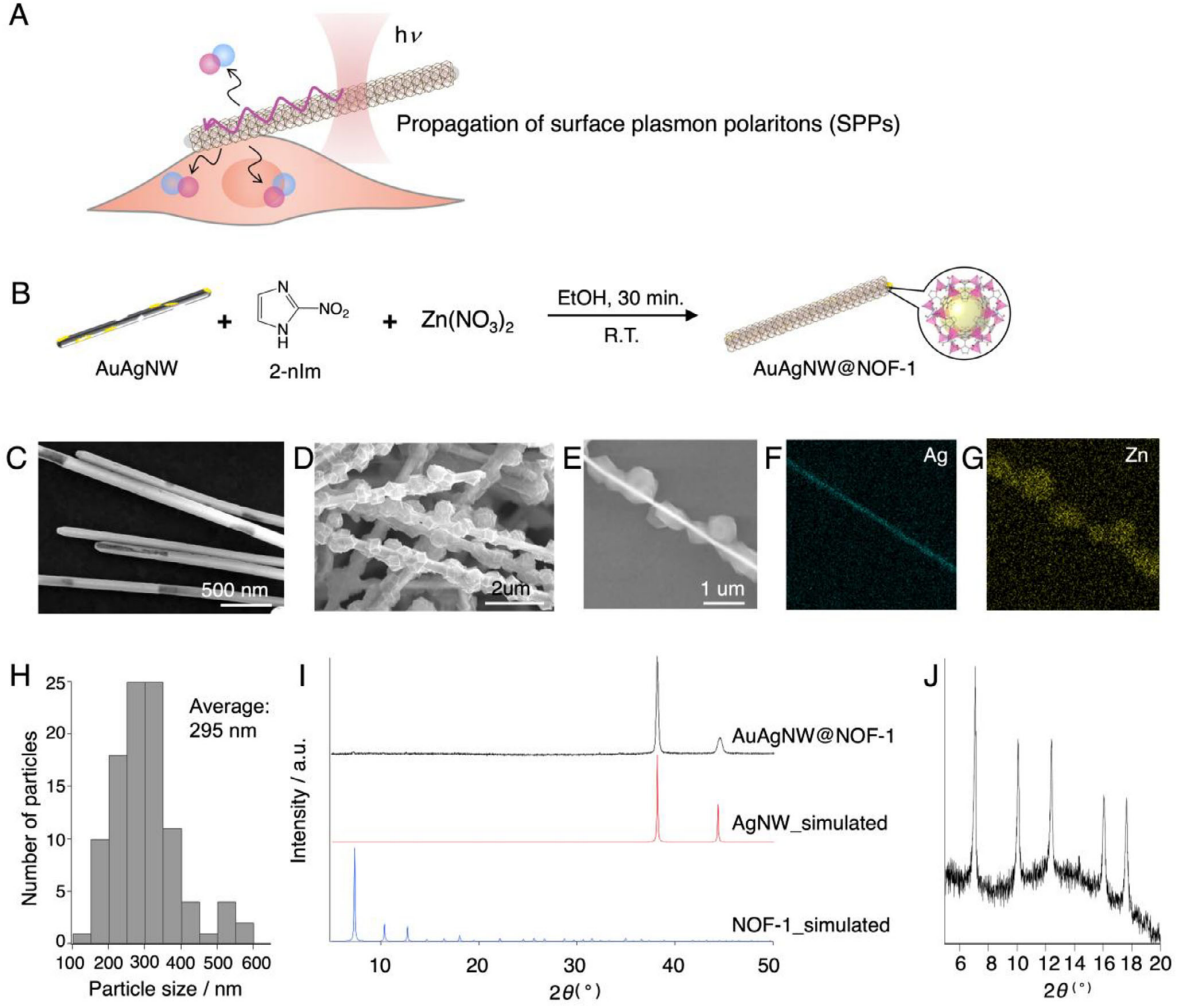
Structural characterization of AuAgNW@NOF‐1. (A) Conceptual illustration of remote‐controlled intracellular delivery of NO by plasmonic waveguide. (B) Schematic illustration of the fabrication process of AuAgNW@NOF‐1. (C) SEM image of AuAgNW. (D) SEM image of AuAgNW@NOF‐1. (E)‐(G) Elemental mapping images of Ag and Zn of AuAgNW@NOF‐1. (H) Particle‐size distribution of NOF‐1 crystals on AuAgNWs analyzed from SEM images. The sizes of 100 randomly selected NOF‐1 crystals were measured using ImageJ. The average particle size was 295 nm ± 89 nm. (I) PXRD pattern of AuAgNW@NOF‐1. Cu Kα radiation (λ = 1.54056 Å). (J) PXRD pattern of AuAgNW@NOF‐1 measured in the 2*θ* range of 5°–20°, with characteristic peaks corresponding to the simulated PXRD pattern of NOF‐1.

We focused on the targeted intracellular delivery of NO by using plasmonic nanowires functionalized with a photo‐responsive, NO‐releasing metal–organic framework, designated as NOF‐1 (Figure 2A) [33]. As a critical signaling molecule in the cardiovascular system, NO plays a vital role in maintaining vascular integrity, regulating smooth muscle proliferation, and modulating cardiac contractility [34, 35]. However, the rapid diffusion and short lifetime of NO present a considerable challenge in elucidating its molecular behavior within the intra/inter‐cellular environment. Therefore, there is a significant demand for innovative tools that offer precise control over the spatiotemporal behaviour of NO gas molecules [33, 36, 37, 38, 39].

To achieve efficient, remote‐controlled NO release, we employed gold‐etched silver nanowires (AuAgNWs). These nanostructures, which we previously developed, feature an increased density of plasmonic coupling points across their surface [26]. Initially, polyvinylpyrrolidone (PVP)‐mediated Ag nanowires (AgNWs) were synthesized by a polyol method (Figure S1) [40]. Subsequently, a galvanic replacement reaction (GRR) was used to generate the AuAgNWs, creating a rough surface morphology that enhances far‐field light coupling to SPPs [26].

The NOF‐1 framework, with the composition of [Zn(2‐nIm)_2_]*_n_*, was then crystallized directly onto the AuAgNW surface by reacting 2‐nitroimidazole (2‐nIm) with zinc nitrate in ethanol at room temperature (Figure 2B). As previously reported for NOF‐1 and related photoresponsive nitro compounds, the proposed NO release pathway involves nitro‐to‐nitrite rearrangement of the 2‐nitroimidazolate ligand, followed by cleavage of the resulting nitrite intermediate to generate NO radicals (Figure S2) [33, 41]. Scanning electron microscopy (SEM) imaging (Figure 2C,D) confirmed the formation of NOF‐1 crystals with particle sizes of several hundred nanometers on the surface of AuAgNWs. Further characterization via energy‐dispersive X‐ray spectroscopy (EDS) mapping of the AuAgNW@NOF‐1 structures clearly identified the presence of zinc, a signature of the NOF‐1 framework, distributed along the nanowire (Figure 2E–G), whereas bare AuAgNW showed no zinc signal (Figure S3). To further clarify the size of the NOF‐1 crystals, we analyzed the particle‐size distribution from SEM images (Figure 2H). The particle size was defined as the longest dimension of each NOF‐1 crystal, and 100 randomly selected crystals were measured. The average particle size was 295 ± 89 nm (mean ± SD, n = 100). Finally, X‐ray diffraction (XRD) analysis revealed characteristic peaks for NOF‐1 at 2*θ* = 7.0° and 12.4° (corresponding to the {110} and {211} planes, respectively), along the signature of AgNW at 2*θ* = 38.1° and 44.5° (corresponding to the {111} and {200} planes, respectively) (Figure 2I,J) [33, 42]. These results confirm the successful synthesis of the AuAgNW@NOF‐1 core‐shell nanostructure.

### 2.2 Light‐Induced NO Release from Bulk MOF‐Coated Nanowires

**FIGURE 3:**
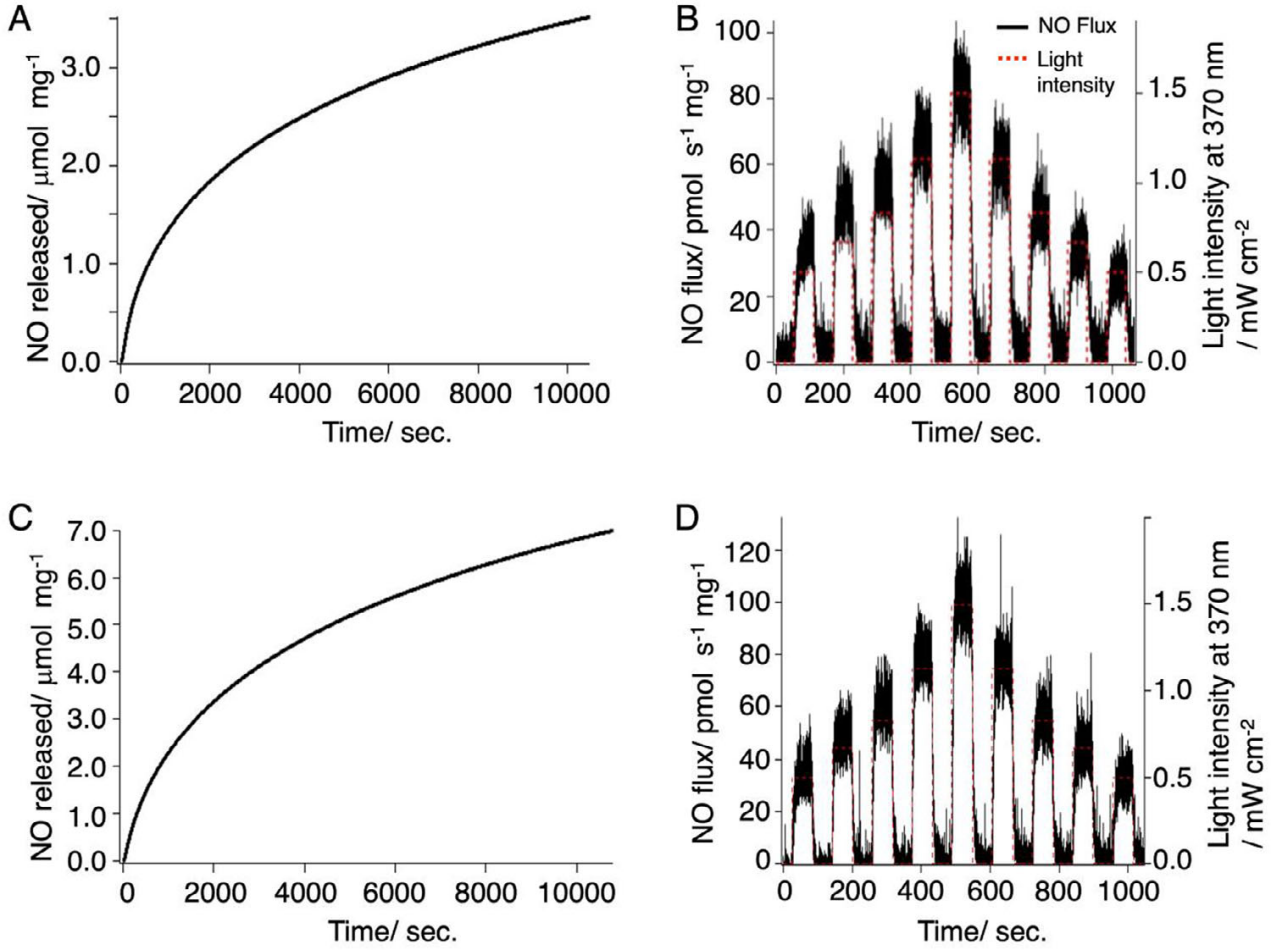
Photo‐induced NO release properties of AuAgNW@NOF‐1 and pristine NOF‐1 powder. (A) NO‐releasing efficiency of AuAgNW@NOF‐1 under continuous 250–385 nm light irradiation (6.4 mW cm^−2^). (B) The NO flux of AuAgNW@NOF‐1 produced under 370 nm light irradiation (band‐pass filter, FWHM: 10 ± 2 nm) as a function of light intensity. (C) NO‐releasing efficiency of NOF‐1 under continuous 250–385 nm light irradiation (6.4 mW cm^−2^). (D) The NO flux of NOF‐1 produced under 370 nm light irradiation (band‐pass filter, FWHM: 10 ± 2 nm) as a function of light intensity.

The NO‐release properties of bulk AuAgNW@NOF‐1 samples under photoirradiation were investigated using an NO‐selective ozone‐chemiluminescence technique (Figure 3A,B). Ultraviolet (UV) irradiation with a wavelength of 250–385 nm was selected, as NOF‐1 exhibits high light‐harvesting efficiency across a broad absorption band between 300 and 400 nm (Figure S6). Under continuous irradiation at a power of 6.4 mW cm^−2^ for 3 h, AuAgNW@NOF‐1 produced more than 3 µmol mg^−1^ of NO (Figure 3A). The observed NO flux from the AuAgNW@NOF‐1 composites is comparable to that of pure NOF‐1 crystals (Figure 3C), indicating that the immobilization process of NOF‐1 on the nanowires does not compromise the NO‐release efficiency of the framework. The slightly larger total amount of NO released from pristine NOF‐1 powder than from AuAgNW@NOF‐1 after 3 h is reasonable because the NO‐release amount was normalized by the total sample mass, which includes the mass of the AuAgNWs in the composite sample. Furthermore, we investigated the photo‐responsive behavior of AuAgNW@NOF‐1 using 370 nm (band‐pass filter, FWHM: 10 ± 2 nm) photoirradiation at varying intensities. As shown in Figure 3B, the composite demonstrated rapid and reversible on‐off switching of NO release over multiple cycles similar to pristine NOF‐1 powder (Figure 3D). Notably, the magnitude of NO flux was directly tunable by modulating the light intensity, confirming the precise remote‐control capability of NO gas afforded by this system.

### 2.3 Plasmon‐Mediated Activation of a Single Nanowire for NO Release

**FIGURE 4:**
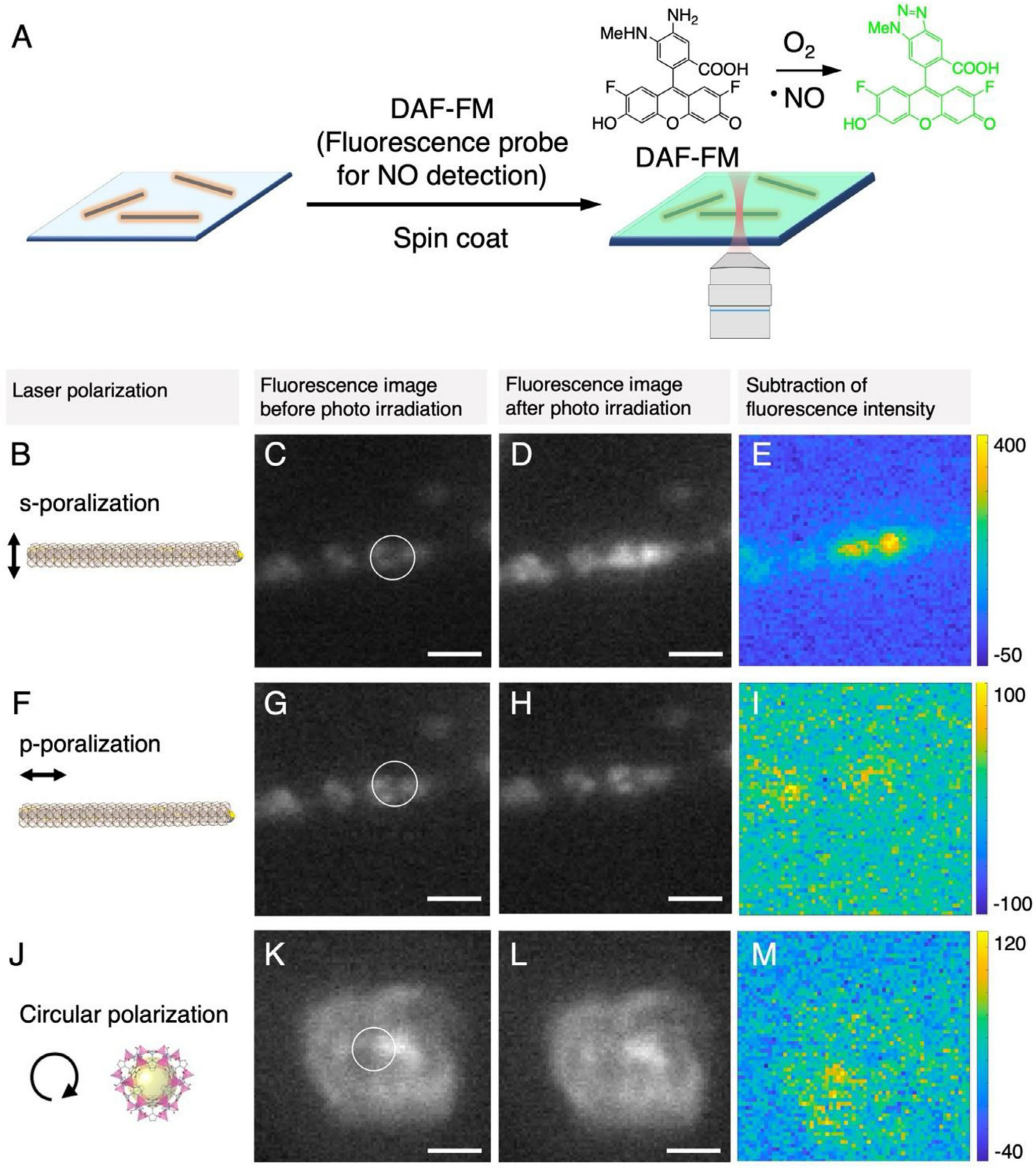
Two‐photon excitation of plasmonic effect on NO release property under 800 nm irradiation (7.6 mW, 10 sec.). (A) Illustration of NO detection scheme after two‐photon excitation. (B) Illustration of the excitation with s‐polarized light. The arrow indicates the polarization direction of the incident laser against the AuAgNW@NOF‐1 composite. (C) Fluorescence image of AuAgNW@NOF‐1 before photo‐stimulation. (D) Fluorescence image of AuAgNW@NOF‐1 after s‐polarized photo‐stimulation. (E) Subtracted image illustrating the change in fluorescence intensity from Figure 4D to Figure 4C. (F) Illustration of p‐polarization excitation against the sample. (G) Fluorescence image of AuAgNW@NOF‐1 before photo‐stimulation. (H) Fluorescence image of AuAgNW@NOF‐1 after p‐polarized photo‐stimulation. (I) Subtracted image illustrating the change in fluorescence intensity from Figure 4H to Figure 4G. (J) Illustration of circular polarization. (K) Fluorescence image of NOF‐1 before photo‐stimulation. (L) Fluorescence image of NOF‐1 after circular‐polarized photo‐stimulation. (M) Subtracted image illustrating the change in fluorescence intensity from Figure 4L to Figure 4K. The white circled area in panels c, g, and k indicates the laser focus position. The scale bars in the images correspond to 2 µm, respectively. A 60x/ 0.95 NA Plan Apo dry objective lens (Nikon) was used for both photo‐stimulation and wide‐field fluorescence imaging. All wide‐field images were acquired using 488 nm excitation light. The color bars in subtraction images represent fluorescence intensity change in units of counts per pixel (counts/ pixel).

Noble metal nanoparticles such as gold are known to catalyze chemical bond cleavage reactions via localized electromagnetic (EM) field enhancement or by the generation of hot carriers [43, 44, 45, 46]. While these existing studies primarily focus on simple organic molecules adsorbed onto particle surfaces, we investigate the influence of the plasmonic properties of AuAgNWs on the reactions of the complex metal–organic frameworks at the single‐nanowire level. In these experiments, AuAgNW@NOF‐1 or NOF‐1 crystals were spin‐coated onto glass coverslips and embedded in a poly(methacrylic acid) (PMMA) matrix containing 0.7 µM diaminofluorescein‐FM (DAF‐FM), a fluorescent NO indicator (Figure 4A). Site‐specific NO release was induced by focused laser irradiation using an optical microscope. Initial experiments using a 375 nm laser confirmed a clear increase in fluorescence intensity at the focal point for both AuAgNW@NOF‐1 composite and NOF‐1 crystals (Figure S7), validating that the system enables visualization of spatially controlled NO release.

To further elucidate the plasmonic contribution, we investigated the photo‐responsive behavior of AuAgNW@NOF‐1 under near‐infrared (NIR) two‐photon excitation. AuAgNWs exhibit plasmonic resonance in the NIR region, which enables strong localized EM enhancement and plasmonic waveguiding effects, where SPPs propagate along the nanowire surface to transport energy beyond the initial excitation site [31, 47, 48, 49, 50, 51, 52]. Figure 4 presents fluorescence images before and after irradiation with 800 nm femtosecond laser pulses. To isolate the plasmonic contribution to NO release from AuAgNW@NOF‐1, we used polarized incident light, as the coupling efficiency of SPPs is highly dependent on the incident polarization. Here, we define s‐polarization as excitation polarization perpendicular to the nanowire's longitudinal axis (Figure 4B), and p‐polarization as parallel to it (Figure 4F). While s‐polarized light (7.6 mW, 10 sec.) induced a significant increase in fluorescence (Figure 4C–E), p‐polarized light under identical conditions yields no observable change (Figure 4G–I). This dependence arises because s‐polarized light couples more efficiently to the plasmon modes at the nanoscale etched sites created during the galvanic replacement reaction. These defect‐like sites act as ‘*hot‐spots*’ that enable momentum matching between incident far‐field light and surface plasmons, resulting in intense local EM enhancement, thereby inducing efficient photo‐cleavage reactions on AuAgNW@NOF‐1. We also observed that increasing laser power leads to fluorescence intensity increase of DAF‐FM, suggesting two‐photon excitation induces NO release (Figure S8). In contrast, pristine NOF‐1 crystals show no significant fluorescence increase under 800 nm excitation (7.6 mW, 10 s) (Figure 4K–M), confirming that the enhanced NO release in the composite system is driven by the plasmonic effect. Note that based on the average particle size of NOF‐1 crystals and the bulk NO‐release amount, the amount of NO released from the laser‐irradiated region of a single AuAgNW@NOF‐1 was roughly estimated to be on the order of sub‐femtomoles (details in Supporting Information). Although some heterogeneity was observed in the fluorescence counts in Figure S8, this likely arises from the non‐uniform distribution of NOF‐1 coverage and the heterogeneous nature of plasmon coupling sites formed during the GRR process.

Interestingly, under s‐polarized excitation, fluorescence intensity increased not only at the laser focal point but also several micrometers away (Figure 4C–E). This indicates that the AuAgNW acts as a plasmonic waveguide; the SPPs propagate along the nanowire, activating the NOF‐1 framework at remote locations. While the adsorption of dielectric MOF crystals attached to the silver nanowire would introduce some attenuation of SPPs propagation, SPPs are known to propagate over 10 µm even with the adsorption of several plasmonic nanoparticles such as AgNPs [31]. Considering the polarization‐dependent fluorescence increase observed under 800 nm excitation together with the absence of a comparable response from pristine NOF‐1 crystals, these results support a plasmon‐mediated contribution to remote‐controlled NO release from AuAgNW@NOF‐1.

### 2.4 Plasmonic Remote‐Controlled NO Release at the Single‐Cell Level

The successful demonstration of the plasmonic waveguiding effect for photo‐induced NO release from AuAgNW@NOF‐1 composite indicates its potential for spatiotemporally controlled intracellular delivery without the need for direct irradiation of the target cell. To further prove this, we integrated the AuAgNW@NOF‐1 composite with the plasmonic nanowire‐based single live‐cell endoscopy developed previously by us [25]. This method uses a 1D plasmonic metal nanowire (∼100 nm in diameter) to facilitate site‐specific access to the cell membrane or intracellular compartments. Here, we demonstrate spatiotemporally controlled NO release onto the membranes of a single individual human aortic smooth muscle cell (HASMC) via remote excitation.

Endoscopic probes were fabricated by attaching AuAgNWs to a sharpened tungsten (W) tip using alternating current (AC) dielectrophoresis (Figure S9B) [25]. Subsequently, the NOF‐1 shell was crystallized onto the AuAgNW surface by immersing the tip in an ethanol solution of Zn(NO_3_)_2_∙6H_2_O and 2‐nIm at room temperature (Figure S9A). SEM imaging shown in Figure S9C confirms the formation of a several‐hundred‐nanometer NOF‐1 shell coating on the probe. To ensure experimental reproducibility and maintain plasmonic performance, freshly prepared AuAgNW@NOF‐1 endoscopic probes were used for each experiment, typically within the same day after preparation. The same probe was not repeatedly used for multiple cell experiments because oxidation of the metallic nanowire surface and possible changes in the NOF‐1 coating may affect plasmon propagation and NO‐release efficiency over time.

**FIGURE 5:**
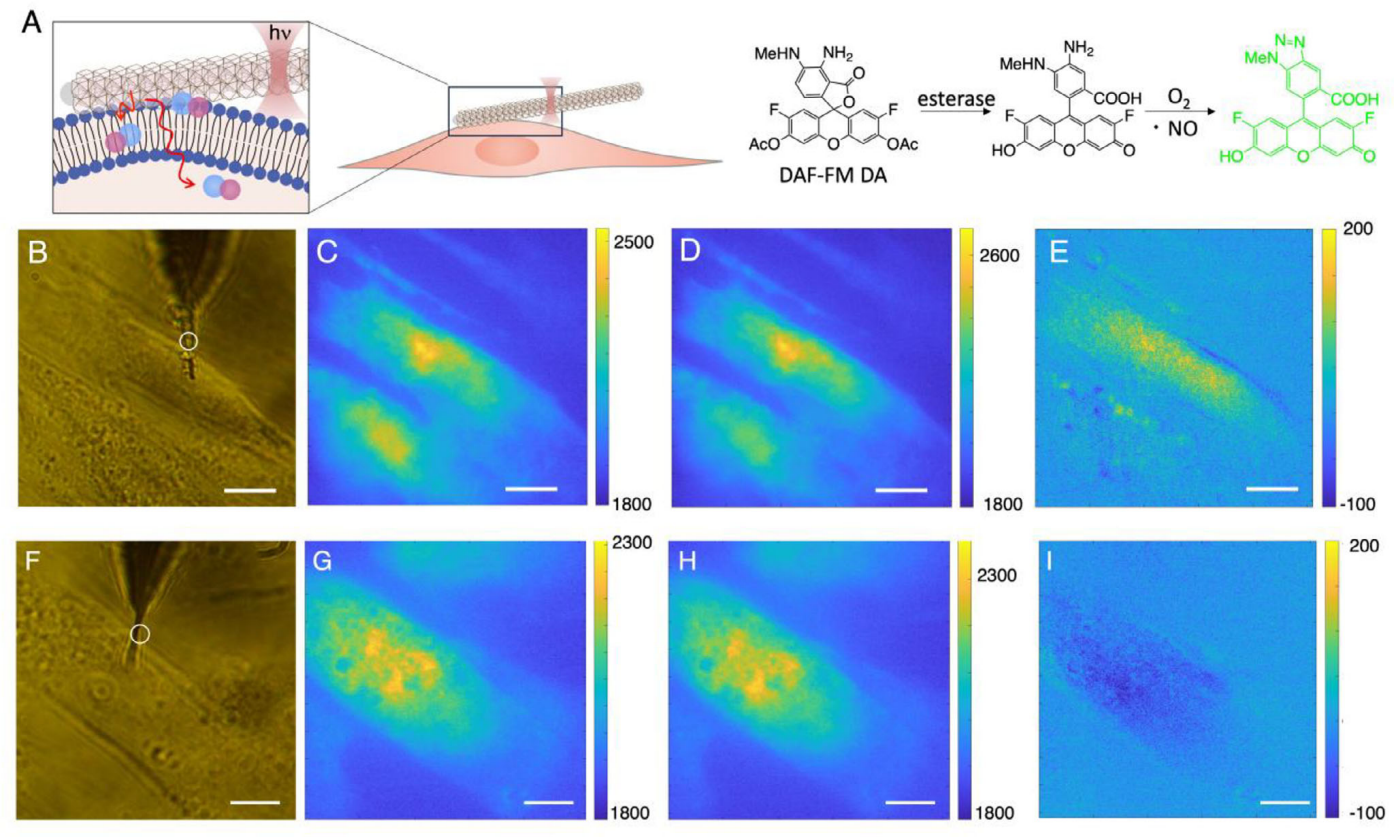
Remote‐controlled NO release on the membrane of a single live human smooth muscle cell under two‐photon excitation (800 nm, 3.6 mW, 7 sec.). (A) Illustration of the remote‐controlled NO release experiment on the cell membrane and the fluorescence reaction of DAF‐FM DA with NO. (B) Transmission image of AuAgNW@NOF‐1 on the cell membrane of a human smooth muscle cell. The white‐circled position indicates the laser focus position for plasmonic remote‐controlled NO release. Note that the focal position is set to be in the extracellular region for the remote‐controlled release. (C) Fluorescence image of the smooth muscle cell stained with DAF‐FM DA before irradiation. (D) Fluorescence image of smooth muscle cells stained with DAF‐FM DA after two‐photon excitation. (E) Subtracted image of fluorescence intensity difference between before and after two‐photon excitation. (F) Transmission image of bare AuAgNW without NOF‐1 coating on the membrane of a human smooth muscle cell. The white‐circled position indicates the laser focus position. (G) Fluorescence image of the smooth muscle cell stained with DAF‐FM DA before irradiation using bare AuAgNW. (H) Fluorescence image of the smooth muscle cell stained with DAF‐FM DA after two‐photon excitation using bare AuAgNW. (I) Subtracted image showing the fluorescence intensity difference between before and after two‐photon excitation using bare AuAgNW. The scale bar of the images indicates 10 µm. A 60x/ 0.95 NA Plan Apo dry objective lens (Nikon) was used for both photo‐stimulation and wide‐field fluorescence imaging. All wide‐field images were acquired using 488 nm excitation light. The color bars in fluorescence images represent fluorescence intensity in units of counts per pixel (counts/ pixel).

Given the high membrane permeability of NO, the AuAgNW@NOF‐1 probe was not inserted into a cell; instead, it was brought into gentle contact with the cell membrane. NO release was remotely triggered by laser irradiation at an extracellular site on the probe (Figure 5A,B). This configuration allows NO to be generated locally at the cell surface and passively diffuse into the cytoplasm. To visualize this delivery, HASMCs were previously stained with DAF‐FM DA, a fluorescent indicator sensitive to intracellular NO. Changes in fluorescence intensity before and after remote excitation of AuAgNW@NOF‐1 were analyzed to verify NO delivery into the target cells. Note that fluorescence of DAF‐FM DA in smooth muscle cells was observed before photo‐stimulation, suggesting the effect of the presence of endogenous NO.

Upon laser irradiation (800 nm, 3.6 mW, 7 sec.), a distinct increase in intracellular fluorescence was observed in the targeted HASMC, while neighboring cells remained unaffected (Figure 5C–E). This result indicates that NO was locally introduced into the target cell through the cell membrane by AuAgNW@NOF‐1, performing site‐specific, remote‐controlled NO release. Additional experiments performed under identical irradiation conditions also showed fluorescence enhancement in a target cell, supporting the reproducibility of remote‐controlled NO release (Figure S11). In contrast, control experiments using bare AuAgNWs (i.e., without NOF‐1 coating) under the same irradiation conditions yielded no fluorescence (Figure 5F–I and Figure S12), confirming that the observed fluorescence increase originated from NO release from the NOF‐1 coating on AuAgNWs. We observed a success rate of approximately 20% (3 out of 15 independent trials). Possible factors contributing to this limited success rate may include heterogeneity in the NOF‐1 coating on AuAgNWs, variability of plasmon coupling points along individual AuAgNWs, and biological variability among cells. Although the current success rate remains limited, the successful trials clearly demonstrate the feasibility of remote‐controlled intracellular photochemistry using plasmonic nanowires. Notably, direct laser focalization on the target cell instead of the remote excitation configuration resulted in a decrease in fluorescence intensity (Figure S13). Although the origin of this decrease is unclear, our previous investigations suggest that direct laser irradiation can alter cellular functions due to light‐induced chemical processes [25] and photothermal effects [53], which affected the fluorescence readout. Ca^2+^ imaging using Ca Green‐1 showed no pronounced increase in fluorescence after remote‐excitation NO release from AuAgNW@NOF‐1 (Figure S14), suggesting that the process did not induce a detectable Ca^2+^‐associated stress response in the target HASMC under these experimental conditions. These findings highlight the advantage of our remote excitation strategy in preserving signal integrity from the cell.

To further evaluate the functional impact of the released NO gas, we monitored changes in intracellular calcium concentration ([Ca^2+^]_i_) in HEK293 cells stably expressing the Ca^2+^ ‐permeable TRPC5 channel, an NO‐activated ion channel [54]. TheFluo‐4, a fluorescent Ca^2+^ indicator, was used to detect changes in intracellular [Ca^2+^]_i_. Upon remote photoactivation of the AuAgNW@NOF‐1 probe, we observed a significant increase in fluorescence intensity, indicative of elevated [Ca^2+^]_i_ (Figure S15). In contrast, no such increase was observed with bare AuAgNW without NOF‐1 (Figure S16). Additionally, such an increase did not occur in TRPC5‐negative HEK293 cells (Figure S17). These results demonstrate that remote‐controlled NO released from AuAgNW@NOF‐1 effectively activates plasma membrane channels, triggering calcium influx and modulating cellular signaling pathways.

## 3 Conclusion

In this study, we successfully synthesized NOF‐1‐coated plasmonic nanowires (AuAgNW@NOF‐1) and demonstrated that the photoresponsive NO release properties of the MOF were preserved upon immobilization on the nanowire surface. We found that plasmonic effects generated on AuAgNWs efficiently induce the photocleavage reaction of NOF‐1, facilitating localized NO release at the nanowire interface. In addition, we characterized a polarization‐dependent NO release efficiency and identified a plasmonic waveguiding effect that enables the local activation of NOF‐1 molecules at a site distal to the laser focal point. This indicates that plasmon‐mediated energy propagation along the nanowire can trigger chemical reactions. These findings significantly advance our understanding of plasmon‐driven chemistry at the nanoscale and highlight the potential of plasmonic nanowire platforms for plasmonic remote‐controlled photochemical activation.

We used these unique plasmonic waveguiding‐induced photochemical reactions to deliver gas molecules into individual target live cells without subjecting them to direct light stimulation. By remotely activating AuAgNW@NOF‐1 in an extracellular environment, we successfully introduced NO into the intracellular environment of an individual single live cell, as evidenced by the increased signal of the NO‐sensitive fluorescence probe. Furthermore, experiments using TRPC5‐expressing HEK293 cells revealed that the remotely released NO triggers [Ca^2+^]_i_ influx, confirming the biological relevance and efficacy of the AuAgNW@NOF‐1 composite. This study demonstrates remote‐controlled photochemistry using AuAgNW@NOF‐1 nanowires as a proof‐of‐concept, in which localized NO release inside a target cell was achieved without direct light stimulation of the cell. While the present demonstration is limited to NO release, this result highlights the potential of plasmonic nanowires as remote activation probes for photoresponsive functional materials and may provide a basis for future single‐cell manipulation strategies.

## 4 Experimental Section

### 4.1 Materials

All chemical reagents were purchased at the highest available commercial quality from Sigma‐Aldrich and Fujifilm Wako Pure Chemical Corporation, and used without further purification.

### 4.2 Synthesis

#### 4.2.1 Synthesis of AgNWs

Silver nanowires were synthesized using a previously reported procedure [40]. Briefly, 9.66 mL of ethylene glycol (EG) was refluxed with 116.3 mg of PVP (0.108 M) at 160°C for 1 h. Next, 80 µL of CuCl_2_ in EG (4 mM) was added to the solution and stirred for 10 min. Afterward, 100 µL of AgNO_3_ in EG solution (0.12 M) was added, causing the solution color to change from transparent to green. Following a 10 min pause, 4.9 mL of AgNO_3_ in EG solution (0.12 M) was added dropwise, at a rate of 100 µL per minute. After all the AgNO_3_ solution had been added, the reaction mixture was further refluxed at 160°C for 1 h. Throughout the reaction, an EG solution containing 0.4% water was used as the solvent. The mixture solution was then washed with isopropanol by centrifuging the solution three times at 1200 rpm for 10 min to remove excess PVP and EG. Finally, the precipitated AgNWs were dispersed in isopropanol.

#### 4.2.2 Synthesis of AuAgNW

AuAgNWs were synthesized following our previously reported procedure [26]. Briefly, 3 mg/mL of pure AgNWs in ethanol (450 µL) and 0.2 mM HAuCl_4_ in aqueous solution (1.8 mL) were mixed in a 15 mL milliQ and stirred for 15 min at 80°C. The obtained nanowires were washed three times with ethanol. Prior to using these AuAgNWs for NOF‐1 coating, they were dispersed in 2 mL of PVP (10 mg/mL) and vortexed for several hours to coat the nanowire surface with PVP. The coated nanowires were then washed three times with ethanol.

#### 4.2.3 Synthesis of AuAgNW@NOF‐1

In a typical synthesis of AuAgNW@NOF‐1, 2‐nitroimidazole (5.66 mg, 0.05 mmol) and Zn(NO_3_)_2_∙6H_2_O (7.45 mg, 0.025 mmol) were dissolved in 3 mL of ethanol. Then, 100 µL of an ethanol dispersion of AuAgNWs (12 mg/mL) was added, and the mixture was stirred for 30 min at room temperature. After the reaction, the resulting materials were washed three times with ethanol.

#### 4.2.4 Synthesis of NOF‐1

2‐nitroimidazole (5.66 mg, 0.05 mmol) and Zn(NO_3_)_2_∙6H_2_O (7.45 mg, 0.025 mmol) were dissolved in 3 mL of ethanol. The solution was stirred at room temperature for 2 h. The color of the solution gradually changed from transparent to white. After the reaction, the resulting crystalline powders were washed three times with ethanol.

### 4.3 Instrumentation and Characterization

#### 4.3.1 Instruments

Scanning electron microscopy (SEM) images were acquired using an SU 5000 (Hitachi High‐Tech). Powder X‐ray diffraction (PXRD) patterns were collected by a SmartLab diffractometer (Rigaku) with Cu Kα radiation ( λ = 1.54056 Å) in Bragg‐Brentano geometry. Ultraviolet–visible (UV–vis) absorption spectra were recorded by a V‐670 spectrophotometer (JASCO).

#### 4.3.2 Photo‐Induced no Release Experiment of Bulk Sample

MeOH suspensions of NOF‐1 and AuAgNW@NOF‐1 were repeatedly spin‐cast (120 r.p.m.) on a round cover slip with a diameter of 12 mm. The weight of the deposited material was accurately determined using a precision scale (typical weight range, 80–120 µg). The samples were placed in a custom‐made cell and irradiated with a 300‐W Xenon lamp (Asahi Spectra Max‐303 equipped with a 250 to 385 nm ultraviolet module and 1.0 collimator lens). A 370 nm band‐pass filter (center wavelength: 370 ± 2 nm; FWHM: 10 ± 2 nm) was used. The incident light power at 370 nm was measured using an Ushio UIT‐201 intensity meter. The NO release measurements were performed using a Sievers NOA 280i chemiluminescence NO analyser. The instrument was calibrated by passing air through a zero filter (Sievers, < 1 ppb NO) and 600 ppb NO gas. The nitrogen carrier gas flow rate was set to 200 mL min‐1 with a cell pressure of 8.5 Torr and an oxygen pressure of 6.1 psig.

#### 4.3.3 Photo‐Induced NO Release Experiment on a Microscope

AuAgNW@NOF‐1 and NOF‐1 were spin‐coated on a 22 × 22 mm^2^ glass substrate at 3000 rpm for 1 min. on cleaned glass cover slips separately. Subsequently, PMMA solution (10 mg/1 mL CHCl_3_) containing DAF‐FM (Funakoshi, 7 µM), 100 uL, was spin coated at 3000 rpm for 1 min on top of the sample. A photo‐induced NO release experiment was performed using a 60x/ 0.95 NA Plan Apo dry objective lens (Nikon).

#### 4.3.4 Fabrication of AuAgNW@NOF‐1 Probe

A tungsten tip of diameter 150 µm was etched by applying a DC voltage, with frequency 50 Hz and amplitude 7 V, between the tungsten wire and a carbon electrode in a 2 M NaOH aqueous solution. An AuAgNW was attached to a tungsten wire by applying alternating current (AC) voltage with a frequency of 1 MHz between the tungsten tip and an electrode in a solution of AuAgNW. Subsequently, the junction between the nanowire and the tungsten tip was glued with epoxy resin. The fixed AuAgNW to a tungsten tip was immersed in a PVP solution (10 mg/mL) for 2 h for PVP modification on the surface of AuAgNW. Finally, the PVP‐modified AuAgNW probe was immersed in a mixture solution of 2‐nitroimidazole and Zn(NO_3_)_2_ in ethanol for NOF‐1 coating.

#### 4.3.5 Cell Culture

The Human Aortic Smooth Muscle cells (HASMCs) were obtained from KURABO (Osaka, Japan) and cultured in Hu‐Media‐SG2 (KURABO), as specified by the manufacturer. The medium composition per 500 mL included the following: recombinant human (rh) FGF‐basic 0.5 mL (2 ng/mL), rh Insulin 0.5 mL (5 µg/mL), rh EGF 0.5 mL (5 ng/mL), fetal bovine serum (FBS) 25 mL (5%), glucose 4.6 × 10^−3^ M, arginine 7.2 × 10^−4^ M, antibiotics 0.5 mL (gentamicin: 50 ug/mL, amphotericin B: 50 ng/mL). Cells were maintained at 37 °C in a humidified atmosphere with 5% CO_2_. The medium was replaced every 2–3 days, and cells were subcultured at ∼80% confluency using trypsin‐EDTA solution.

The HEK293 and HEK293‐TRPC5 cells were cultured in medium consisting of DMEM supplemented with 10% (v/v) FBS and 1% (v/v) penicillin/streptomycin in a humidified incubator (37°C, 5% CO_2_). The medium was changed every 2–3 days. The cells were passaged with trypsin‐EDTA solutions. For the HEK293‐TRPC5 cells, 100 µg mL^−1^ G418 was added to the culture medium for their selection.

#### 4.3.6 Cell Staining for Wide‐Field Fluorescence Imaging

Cultured human aortic smooth muscle cells (HASMCs) were stained with 1 mL of DAF‐FM DA solution (5 µM in PBS buffer) and incubated for 5 min. After incubation, the cells were washed three times with PBS buffer and then immersed in HBSS buffer.

For Ca^2+^ imaging to evaluate cellular response to NO release from AuAgNW@NOF‐1, HASMCs were stained with 1 mL Ca Green‐1 solution (5 µM in PBS buffer) and incubated for 10 min. After incubation, the cells were washed three times with PBS buffer and then immersed in HBSS buffer.

For HEK293 and HEK293‐TRPC5 cells, 1 mL of Fluo‐4 AM solution (1 µM in PBS buffer) was added, followed by a 15 min incubation. Afterward, the cells were washed three times with PBS buffer and subsequently immersed in HBSS buffer.

#### 4.3.7 NO Release on a Target Single Live‐Cell Membrane

1 mL of DAF‐FM DA solution (Funakoshi, 5 µM in PBS) was added to a glass‐bottom dish containing cultured HASMC and incubated for 5 min. After the incubation, the cells were washed three times with PBS and then immersed in HBSS. The glass‐bottom dish was placed on an inverted microscope (Ti‐U Nikon). The AuAgNW@NOF‐1 probe was gently put on the cell membrane. The movements of the probe were controlled by a motorized four‐axis micromanipulator (PCS‐6000CR, Thorlabs) with a stepper motor and piezoelectric translation, which allowed for precise access of the probe to the cell membrane of a live HASMC. For NO release, an 800 nm femtosecond pulsed laser (120 fs, 80 MHz) was focused on the AuAgNW@NOF‐1 after the probe was placed at the target location, and a fluorescence image of a live cell was collected before and after the laser irradiation. A 60x/ 0.95 NA Plan Apo dry objective lens (Nikon) was used for both photo‐stimulation and wide‐field fluorescence imaging.

## Author Contributions

T.I., S.F. conceived the project and directed the research. T.I., J.L., J.T., S.T., and K.H. performed experiments associated with material synthesis and characterization. T.I. and E.S.G conducted the NO gas detection by an NO analyzer. T.I. and H.U. conducted the two‐photon excitation of NO release experiment and data analysis. T.I., N.T., and Y.M performed cell experiments. T.I. and S.F. wrote the manuscript. All authors discussed the results and commented on the manuscript.

## Funding

JST PRESTO No. JPMJPR2104, JSPS KAKENHI No. JP23H01803, JP23K26496, JP 23J04877, JP21H04634, JP24K21713, JP25H00827, JP23H04877, JP25K22237, JP26K01345, JP21K18192, JP26H00725, JP26H02217. Research Foundation of Flanders (FWO) research grant (G022724N), KU Leuven (C14/23/090), Toyota Riken Scholar, Cooperative Research Program of “Network Joint Research Center for Materials and Devices (Mext)” (Grant No. 20261051, 20251024).

## Conflicts of Interest

The authors declare no conflicts of interest.

## Supporting information




**Supporting File**: smll74836‐sup‐0001‐SuppMat1.docx.

## Data Availability

The data that support the findings of this study are available from the corresponding author upon reasonable request.
