# Supplementary material for: Remote‐Controlled Release of Nitric Oxide by Plasmonic Waveguide Coated With Metal–Organic Frameworks
Source: Small. 2026 Jul 31;22(53):e74836. doi: 10.1002/smll.74836 (PMC13592403; doi:10.1002/smll.74836)
Supplement: Supplementary file 1 [file SMLL-22-e74836-s001.docx]

**Supporting Information**

Remote-controlled release of nitric oxide by plasmonic waveguide coated with metal-organic frameworks

Tomoko Inose,^1,2,3,4^* Javier Lopez-Cabrelles,^1^ Javier Troyano,^1^ Shun Tokuda,^1^ Elí Sánchez-González,^1^ Nobuaki Takahashi,^4^ Yasuo Mori,^4^ Kenji Hirai,^5^ Hiroshi Uji-i^5,6^ and Shuhei Furukawa^1,4^

^1^Institute for Integrated Cell-Material Sciences (WPI-iCeMS), Kyoto University, iCeMS Research Bldg, Yoshida, Sakyo-ku, Kyoto 606-8501, Japan

^2^The HAKUBI Center for Advanced Research, Kyoto University, Yoshida, Sakyo-ku, Kyoto 606-8501, Japan

^3^JST-PRESTO, Saitama 332-0012, Japan

^4^Department of Synthetic Chemistry and Biological Chemistry, Graduate School of Engineering, Kyoto University, Katsura, Nishikyo-ku, Kyoto 615-8510, Japan

^5^Research Institute for Electronic Science (RIES), Hokkaido University, N20W10, Sapporo 001-0020, Japan

^6^KU Leuven, Departement Chemie, Celestijnenlaan 200F, 3001 Heverlee, Belgium

**Functionalization of NOF-1 on nanowires**

**Figure S1. SEM image of AgNW**.

**Figure S2. Photochemical nitro-to-nitrite rearrangement of the 2-nitroimidazole ligands.**

**Figure S3. SEM image and elemental mapping images of Ag and Zn of AuAgNW.**

**Figure S4. SEM image of NOF-1.**

**Figure S5. The PXRD patterns of NOF-1.**

**Figure S6. UV-vis diffuse-reflectance spectrum of NOF-1 powders.**

**Photo-induced NO release properties of single AuAgNW@NOF-1**

**Figure S7.** **Fluorescence images of** (**A**) AuAgNW@NOF-1 before photo-irradiation, (**B**) AuAgNW@NOF-1 after photo-irradiation, (**C**) NOF-1 before photo-irradiation, and (**D**) NOF-1 after photo-irradiation. A 375 nm continuous-wave (CW) laser was used to induce photo-stimulated NO release reactions from both AuAgNW@NOF-1 and NOF-1. Wide-field fluorescence imaging was performed using an excitation wavelength of 488 nm to visualize the fluorescence of DAF-FM. The scale bar of all images is 10 μm.

**Figure S8.** **Dependence of NO release efficiency on polarization during 800 nm two-photon laser irradiation of AuAgNW@NOF-1.** The subtraction of fluorescence intensity represents the change in fluorescence intensity of DAF-FM before and after irradiation, where larger values indicate higher NO release efficiency. Regardless of laser power intensity, NO release efficiency from AuAgNW@NOF-1 was observed to be higher under s-polarization compared to p-polarization irradiation. These results confirm that the NO release efficiency of NOF-1 on AuAgNW exhibits polarization dependence. The larger error bars observed for s-polarization are attributed to the heterogeneity of the NOF-1 coating and the uneven distribution of plasmon coupling points on the nanowire. In contrast, circularly polarized light at 800 nm applied to NOF-1 alone did not result in efficient NO release compared to AuAgNW@NOF-1.

**Estimation of NO release from NOF-1 crystals within the laser-irradiated region**

The amount of NO release from NOF-1 crystals within the laser-irradiated region was roughly estimated usign the particle-size distribution of NOF-1 crystals on AuAgNWs and the bulk NO-release amount of AuAgNW@NOF-1.

The average particle size of NOF-1 crystals on AuAgNWs was determined to be 295 nm from SEM images (Fig. 2H). The laser spot diameter was assumed to be approximately 1 μm based on diffraction limited focusing of an 800 nm laser through a 60x/0.95 NA objective lens.

For this estimation, NOF-1 crystal were assumed to be spherical, and the laser irradiated area was assumed to be fully covered with NOF-1 crystals. The number of NOF-1 crustals within the irradiated area was estimated from the ratio of the laser spot ares to the projected area of a single NOF-1 crystal:

N = (D_laser / d)^2^

where D_laser is the laser spot diameter and d is the average particle diameter of NOF-1. Using D_laser = 1.0 μm and d = 295 nm, the number of NOF-1 crystals within the irradiated area was estimated to be approximately 12.

The volume of a single NOF-1 crystal was estimated by assuming a spherical shape:

V_particle = 4/3π(d/2)^3^

Using d = 295 nm, the volume of one NOF-1 crystal was estimated to be approximately 1.35 × 10^-14^ cm^3^. The mass of one NOF-1 crystal was then calculated using the crystallographic density of NOF-1: 1.174 g cm^-3^. ^33^

m_particle = ρV_particle

This gives an estimated mass of approximately 1.59 × 10^-14^ g per NOF-1 crystal.

Therefore, the total mass of NOF-1 within the laser-irradiated region was estimated to be approximately 1.9 × 10^-13^ g, corresponding to 1.9 × 10^-10^ mg.

Finally this estimated NOF-1 was multiplied by the bulk NO-release amount of AuAgNW@NOF-1 (Fig. 3). Using a bulk NO-release amount of approximately 3.5 μmol mg^-1^, the amount of NO released within the laser-irradiated region was estimated to be approximately 6.7 × 10^-16^ mol. Thus, the NO release per laser excitation event was estimated to be on the order of 10^-16^ mol, corresponding to sub-femtomoles.

This value should be regarded as an approximate estimate based on simplifying assumptions rather than an absolute intracellular NO concentration because the calculation assumes full coverage of the irradiated area by NOF-1 crystals and applies the bulk NO-release capacity to the 800 nm plasmon-mediated excitation condition.

**Endoscopy experiment**

**Figure S9 Fabrication of nanowires for endoscopy experiment.** (**A**) Schematic illustration of a single AuAgNW coated with NOF-1 for the intracellular NO release experiment. (**B**) SEM image of AuAgNWs attached to a W wire. (**C**) SEM image of AuAgNW@NOF-1 attached to a W wire.

**Figure S10 The effect of photo-irradiation on NOF-1 on AuAgNWs.** (**A**) Transmission image of a single AuAgNW@NOF-1 before photo-irradiation at the yellow-circled area (800 nm,3.6 mW, 7 sec.). (**B**) Transmission image of the same AuAgNW@NOF-1 after photo-irradiation, showing that NOF-1 remains on the AuAgNW. The scale bar of all images is 10 μm.

**Figure S11. NO release on the membrane of a single live human smooth muscle cell under remote two-photon 800 nm irradiation (3.6 mW, 7 sec.).** (**A**), (**E**) Transmission image of AuAgNW@NOF-1 on the cell membrane of a human smooth muscle cell. The white-circled position indicates the laser focus position for remote-controlled NO release. (**B**), (**F**) Fluorescence image of the smooth muscle cell before two-photon irradiation, stained with DAF-FM DA. (**C**), (**G**) Fluorescence image of smooth muscle cell after two-photon irradiation, stained with DAF-FM DA. (**D**), (**H**) Subtraction image of fluorescence intensity difference between after and before two-photon irradiation. A slight increase in fluorescence intensity of a target cell was observed. The scale bar in all images is 10 μm. A 60x/ 0.95 NA Plan Apo dry objective lens (Nikon) was used for both photo-stimulation and wide-field fluorescence imaging. All wide-field images were acquired using 488 nm excitation light. The color bars in fluorescence images represent fluorescence intensity in units of counts per pixel (counts/ pixel).

**Figure S12.** **Control experiment of intracellular NO delivery using AuAgNW on the membrane of a single live human smooth muscle cell under two-photon 800 nm irradiation (3.6 mW, 7 sec.).** (**A**) Transmission image of AuAgNW on the cell membrane of a human smooth muscle cell. The white-circled position indicates the laser focus position for remote-controlled NO release. (**B**) Fluorescence image of the smooth muscle cell before two-photon irradiation, stained with DAF-FM DA. (**C**) Fluorescence image of smooth muscle cell after two-photon irradiation, stained with DAF-FM DA. (**D**) Subtraction image of fluorescence intensity difference between after and before two-photon irradiation. Slight fluorescence quenching was observed instead of an increase in fluorescence intensity. The scale bar in all images represents 10 µm. A 60x/ 0.95 NA Plan Apo dry objective lens (Nikon) was used for both photo-stimulation and wide-field fluorescence imaging. All wide field images were acquired using 488 nm excitation light. The color bars in fluorescence images represent fluorescence intensity in units of counts per pixel (counts/ pixel).

**Figure S13.** **Effect of direct photo-stimulation to a target single live human smooth muscle cell at two-photon 800 nm irradiation (3.6 mW, 7 sec.).** (**A**) Transmission image of AuAgNW@NOF-1 on the cell membrane of a human smooth muscle cell. The white-circled position indicates the laser focus position for remote-controlled NO release. (**B**) Fluorescence image of the smooth muscle cell before two-photon irradiation, stained with DAF-FM DA. (**C**) Fluorescence image of smooth muscle cells after two-photon irradiation, stained with DAF-FM DA. (**D**) Subtraction image of fluorescence intensity difference between after and before two-photon irradiation. A decrease in fluorescence intensity of a target cell was observed. The scale bar of all images is 10 μm. A 60x/ 0.95 NA Plan Apo dry objective lens (Nikon) was used for both photo-stimulation and wide-field fluorescence imaging. All wide-field images were acquired using 488 nm excitation light. The color bars in fluorescence images represent fluorescence intensity in units of counts per pixel (counts/ pixel).

**Figure S14.** **Ca²⁺ imaging of a target single live human smooth muscle cell during remote-excitation NO release from AuAgNW@NOF-1 under two-photon 800 nm irradiation. (3.6 mW, 7 sec.).** (**A**), (**E**) Transmission image of AuAgNW@NOF-1 on the cell membrane of a human smooth muscle cell. The white-circled position indicates the laser focus position for remote-controlled NO release. (**B**), (**F**) Fluorescence image of the smooth muscle cell before two-photon irradiation, stained with Ca Green-1. (**C**), (**G**) Fluorescence image of smooth muscle cells after two-photon irradiation, stained with Ca Green-1. (**D**), (**H**) Subtraction image of fluorescence intensity difference between after and before two-photon irradiation. A decrease in fluorescence intensity of a target cell was observed. The scale bar of all images is 10 μm. A 60x/ 0.95 NA Plan Apo dry objective lens (Nikon) was used for both photo-stimulation and wide-field fluorescence imaging. All wide-field images were acquired using 488 nm excitation light. The color bars in fluorescence images represent fluorescence intensity in units of counts per pixel (counts/ pixel).

**Figure S15. NO release on the membrane of a single TRPC5-HEK293 cell under remote two-photon 800 nm irradiation (1.2 mW, 10 sec.).** (**A**) Transmission image of AuAgNW@NOF-1 on the cell membrane of a TRPC5-HEK293 cell. The white-circled position indicates the laser focus position for remote-controlled NO release. (**B**) Fluorescence image of the TRPC5-HEK293 cell before two-photon irradiation, stained with Fluo-4 AM. (**C**) Fluorescence image of TRPC5-HEK293 cell after two-photon irradiation, stained with Fluo-4 AM. (**D**) Subtraction image of fluorescence intensity difference between after and before two-photon irradiation. A slight increase in fluorescence intensity of a target cell was observed. The scale bar of all images is 10 μm. A 60x/ 0.95 NA Plan Apo dry objective lens (Nikon) was used for both photo-stimulation and wide-field fluorescence imaging. All wide-field images were acquired using 488 nm excitation light. The color bars in fluorescence images represent fluorescence intensity in units of counts per pixel (counts/ pixel).

**Figure S16. Control experiment of NO release on the membrane of a single TRPC5-HEK293 cell using AuAgNW under remote two-photon 800 nm irradiation (1.2 mW, 10 sec.).** (**A**) Transmission image of AuAgNW on the cell membrane of a TRPC5-HEK293 cell. The white-circled position indicates the laser focus position for remote-controlled NO release. (**B**) Fluorescence image of the TRPC5-HEK293 cell before two-photon irradiation, stained with Fluo-4 AM. (**C**) Fluorescence image of TRPC5-HEK293 cell after two-photon irradiation, stained with Fluo-4 AM. (**D**) Subtraction image of fluorescence intensity difference between after and before two-photon irradiation. The scale bar in all images is 10 μm. A 60x/ 0.95 NA Plan Apo dry objective lens (Nikon) was used for both photo-stimulation and wide-field fluorescence imaging. All wide-field images were acquired using 488 nm excitation light. The color bars in fluorescence images represent fluorescence intensity in units of counts per pixel (counts/ pixel).

**Figure S17. Control experiment of NO release on the membrane of a single HEK293 cell under remote two-photon 800 nm irradiation (1.2 mW, 10 sec.).** (**A**) Transmission image of AuAgNW@NOF-1 on the cell membrane of a HEK293 cell. The white-circled position indicates the laser focus position for remote-controlled NO release. (**B**) Fluorescence image of the HEK293 cell before two-photon irradiation, stained with Fluo-4 AM. (**C**) Fluorescence image of HEK293 cell after two-photon irradiation, stained with Fluo-4 AM. (**D**) Subtraction image of fluorescence intensity difference between after and before two-photon irradiation. The scale bar in all images is 10 μm. A 60x/ 0.95 NA Plan Apo dry objective lens (Nikon) was used for both photo-stimulation and wide-field fluorescence imaging. All wide-field images were acquired using 488 nm excitation light. The color bars in fluorescence images represent fluorescence intensity in units of counts per pixel (counts/ pixel).
